# Kinetic-based determinations in continuous-flow analysis

**DOI:** 10.1155/S1463924686000366

**Published:** 1986

**Authors:** M. D. Luque de Castro, Miguel Valcárcel

**Affiliations:** Department of Analytical Chemistry Faculty of Sciences University of Córdoba Córdoba Spain


Journal of Automatic Chemistry/*Journal of Clinical Laboratory Automation*, Vol. 8, No. 4 (October–December 1986), pp. 186–192

### Kinetic-based determinations in continuous-flow analysis

**M. D. Luque de Castro and Miguel Valcárcel**

*Department of Analytical Chemistry, Faculty of Sciences, University of Córdoba, Córdoba, Spain*

### Introduction

The automation of analytical processes is becoming an increasingly important area in analytical chemistry and is a response to the growing complexity of the problems faced and to the need to cut down on expenses on analytical control.

If, in general, it is important to replace the human in laboratory work, then the automation of processes based on dynamic measurements is essential in order to avoid both determinate and indeterminate errors. Presently, it is common to automate one or more of the steps in the analytical process—preliminary operations, signal measurement, and data acquisition and treatment—by kinetic measurements.

Despite their significance, the kinetic aspects of analytical chemistry have not been dealt with systematically to date [1, 2]. Kinetics has influenced analytical chemistry in two ways. First, it is as important as thermodynamics and should be strictly taken into account in optimizing analytical procedures. On the other hand, the dynamic aspects are the basis for kinetic methods of analysis, in which measurements are carried out in a physical, chemical or physico-chemical kinetic state. The transient character of the signal obtained may arise from the dynamics of the system under study (for instance chemiluminescence techniques) or that imposed by the technique (for example 'chrono' electroanalytical techniques), or, alternatively, may be inherent in the particular methodology (atomic absorption spectroscopy with electrothermal vaporization).

Kinetic determination, also referred to as 'reaction-rate methods', is a particular type of kinetic method and is based on the measurement of the rate of a chemical reaction which is related to the analyte concentration. The kinetic character this kind of determination is imposed by the system; the methodology and instrumentation used must conform to the dynamic situation.

Of the three types of automatic analysers currently available (discrete, continuous and robotic), continuous analysers have the greatest kinetic character. Typical transient signals are obtained as a result of the physical characteristics of continous-flow configurations. Kinetic variables, such as the flow rate, exert a significant influence. So continuous-flow methods can be considered as kinetic methods. In the case of flow injection analysis, detection is doubly dynamic in nature: neither physical (homogenization of a sample zone) nor chemical equilibrium is generally attained.

This paper deals with kinetic determinations based on reaction-rate measurements carried out in automatic continuous-flow analysers. Applications of this type of automatic method are generally based on the relationship between the signal (rather than the reaction rate) and the analyte concentration.

### General features of kinetic determinations

There are a series of characteristics which clearly distinguish kinetic from thermodynamic or equilibrium methods; essentially this is the strict control required of such variables as time and temperature, measurement type, selectivity and sensitivity.

Time as a variable must be strictly controlled in every operation carried out in applying a kinetic method. Measurement time is not the only key factor to control; the duration of the preliminary operation is equally important and its influence on the data acquisition system must be known.

Temperature is a critical variable in kinetic methods because it affects the kinetics of the reaction—if the constant is sufficiently large—more than does its equilibrium. With the equilibrium methods, slight changes in temperature will not have a dramatic influence; kinetic methods require precise control of temperature within 0·001 to 0·1 °C.

An important feature of kinetic methods is that they are based on relative measurements of the signal, so that its initial value (absorbance, fluorescence, potential etc.) has no influence. Only the rate of change of the signal, which is not influenced by turbidity, initial coloration or fluorescence, dirty cell, junction potentials and so on has to be measured. A large number of potential interferents is thus eliminated, thereby increasing selectivity. Other reasons for the selectivity of kinetic methods being better than that of equilibrium methods is that differential rates are used to isolate information for a particular analyte; also analytical data is provided quickly and can therefore be applied to slow reactions. Such reactions would be impractical for equilibrium measurements, especially if side reactions were involved.

Kinetic-based determinations are known to be very sensitive. Reliable results can be obtained in the parts per thousand million and parts per million ranges for many constituents. Even though the magnitude of the parameter measured is usually larger for equilibrium methods than for rate methods, the latter are often more reliable for low analyte concentrations in real samples. The reason for this is again the advantage of relative measurements as compared to absolute.

*Table 1. General types of kinetic determinations.*

According to:

1. Type of reaction
   - Catalysed
     - enzymatic
     - non-enzymatic
   - Uncatalysed
2. Half-life of the reaction
   - Ultrafast
   - Fast
   - Intermediate
   - Slow
3. Experimental methodology
   - Closed systems
   - Open systems
4. Data manipulation methodology
   - Determination of single species (catalysed reactions)
     - Differential methods
       - initial rate
       - fixed time
       - variable time
     - integral methods
       - tangent
       - fixed time
       - variable time
   - Differential kinetic determinations
     - Graphical methods
       - logarithmic extrapolation
       - linear graphical extrapolation
     - Mathematical methods: proportional equations

One of the most important limitations of the technique is imposed by the reaction rate itself. The half-life of the reaction must be greater than the mixing time, which is a function of the instrumentation available—the instruments must be suited to the reaction rate. Nevertheless, very slow reactions with half-lives of over a few hours are not practical for routine analysis as they may require several minutes to obtain reliable rate data.

The last step of analytical processes based on measurements under non-equilibrium conditions is of great importance in contrast with methodologies applied at equilibrium. Not only must the detector response be fully constant over time; it is also necessary to have a system for fast acquisition of pairs signal-time data, as well as to treat such data in a suitable manner in order to be able to relate the reaction rate to the analyte concentration. To avoid introducing human errors at this stage, electronic ratemeters were used in the past; today, these have been replaced with an on-line microcomputer.

### Classification of reaction rate methods

Kinetic determinations can be classified in several ways: see table 1. A first distinction may be made according to the characteristics of the reaction being monitored. Catalysed reactions (both enzymatic and non-enzymatic) account for over 90% of kinetic determinations. Most of these reactions are of a redox nature. The analyte may be the catalyst, the substrate or a modificator (activator or inhibitor).

The half-life of a reaction is a datum of great interest to which the methodology used must be suited. It is possible to distinguish between ultra-fast (half-life $10^{-6}$–$10^{-2}$ s), fast ($10^{-2}$–10 s), intermediate (10–100 s) and slow reactions (minutes–hours).

Depending on how they are applied, kinetic determinations can be classified by considering the influence of external experimental variables on the reaction. In the so-called closed systems, these variables are kept constant, mixing is effected in a cell and the signal is monitored as a function of time. Open systems, which involve such external factors as the flow-rate or abrupt changes in temperature, pressure, or electrical field, include continuous mixing, relaxation and stat methods.

How the pairs of signal-time data will be treated in order to relate them to the analyte concentration depends on whether they are used to determine a single analyte or a mixture of two or more analytes (kinetic-differential methods).

The determination of a single species in catalysed reactions can be carried out by differential or integral methods. In the former, data are collected at the onset of the reaction, when changes in the concentration of reactants are negligible and the reaction can be considered of pseudo-zeroth order. In such cases, the initial rate is directly proportional to the concentration of the catalyst. Alternatively, measurements of the increase in the signal over a fixed interval, or the increase in the time needed to attain a fixed signal level, can be performed.

Integral methods are applied when data are taken throughout the kinetic curve, so the simplification described previously cannot be made. They can be of pseudo-first or pseudo-second order with respect to one of the reactants and can also be implemented by measuring the reaction rate through the value of the tangent, a signal increment of a time increment.

Kinetic-differential methods are based on the different reaction rate of two or more analytes with a common reagent, both of which yield similar products which are simultaneously monitored. Graphical methods are based on the use of the whole kinetic curve and almost always require knowing the value of the signal at equilibrium. The proportional equation method is simpler; it is based on measurements of the signal at two or more times under different experimental conditions. Systems of $n$ equations in $x$ unknowns are established ($n > x$). It is unnecessary to plot all of the kinetic curve or all of the data collected in this case.

### Classification of continuous-flow analytical methods

Table 2 is a broad classification of continuous methods. A distinction is made between the following techniques:

*Segmented methods* are characterized by sequential aspiration of the samples, between which air bubbles are introduced to avoid cross-contamination. They derive from the scheme proposed by Skeggs in 1957 [3] and have been widely commercialized, especially for use in clinical chemistry.

*Nonsegmented methods* allow for further division according to whether or not there is any sample injection. When injection is involved, the sample can be inserted into a continuous carrier or reagent stream (flow injection analysis) of, alternatively, fast injection simultaneously with the reagent, and stopping the mixture at the detector (stopped-flow methods). The method with no sample injection can be applied to discrete samples which are aspirated over an interval until signal equilibrium is attained; or to systems controlled in a continuous manner by aspiration, mixing with the reagent(s) and continuous recording of the signal provided by the unknown (Completely Continuous Flow Analysis—CCFA). Of all these, only the last type is not suitable for performing kinetic measurements.

### Continuous kinetic-based methods

Among the different types of methods commented on, only the classical stopped-flow methods are applicable to fast reactions (half-lives shorter than 10 s); the remainder (SFA and UFA) are only used for slow reactions.

*Table 2. Continuous-flow analytical methods.*

| | | |
|---|---|---|
| 1. Air-segmented | | |
| 2. Non segmented | With injection | FIA |
| | | stopped-flow |
| | Without injection | sample aspiration |
| | | CCFA |

A general segmented flow configuration for kinetic measurements is shown in figure 1. The propelling system (usually a peristaltic pump) continuously moves the segmented sample and reagent streams, which come into contact either through a dialysis membrane, if analyte separation is required, or on a confluence point, if no separation is needed. The reactions start somewhere along the thermostatted reactor, which directs the sample to two detection cells in series; the sample in turn provides a signal as it passes through each of the cells. The time between both measurements (delay time) is dictated by the characteristics of the reactor between the cells, as well as by the flow-rate provided by the propelling system. The air bubbles, characteristic of these methods, can either (*a*) be removed by means of a debubbler located immediately before the first flow-cell; or (*b*) pass through the detection system (SMAC autoanalyzer), which is programmed to eliminate unwanted signals. A more complicated scheme than that of figure 1 is included in the Technicon SMAC methodology for the determination of the activity of glutamic pyruvic transferase by a three-point rate reaction. Two thermostatted coils placed between the three cells and the selection of measurement time intervals is based on well-defined conditions under which the enzyme behaviour conforms to a zeroth-order kinetics.

Unsegmented Flow Methods, UFA, offer a wider range of kinetic measurements. A distinction can be made depending on whether the sample is or not inserted into the system by injection (Flow Injection Analysis—FIA, and stopped-flow methods) or by aspiration (other methods).

Kinetic methods have been developed in FIA with the aid of very different configurations (figures 2 and 3) of outstanding simplicity—especially those requiring the use of a single detection point. The most generic modifications introduced into an FIA configuration to obtain several signals with a single detector in a single injection operation are shown in figure 2, in which they have been classified according to the absence (*a*, *b*, *c*) or presence (*d*) of splitting and/or confluence point(s): (*a*). An abnormally large sample bolus which gives rise to two reaction zones (two signals) at the two carrier-sample interfaces [4 and 5]. A similar configuration involving injected volumes typical of FIA, and two cells located in series in the FIA configuration and aligned with the optical path of the detector can also be utilized. The reactor connecting both cells determines the delay time in this case as well. (*b*) Two series injection valves which

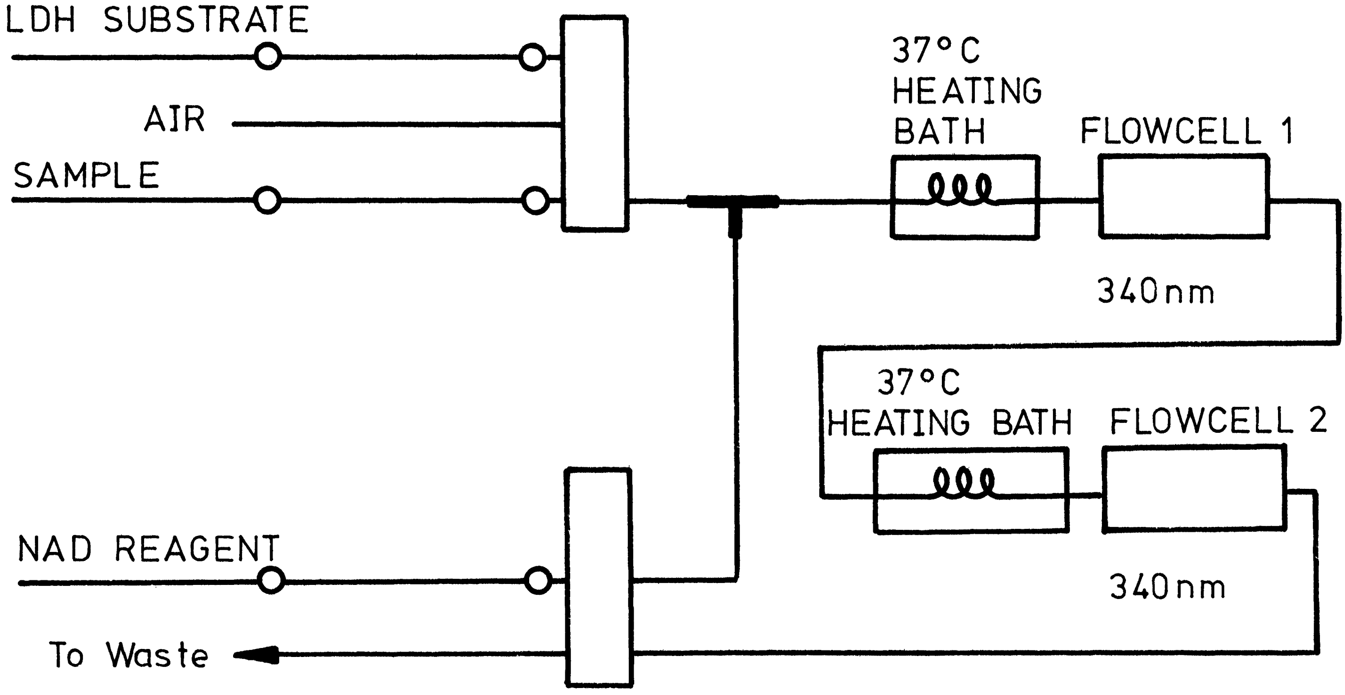

*Figure 1. Basic configuration for kinetic determinations of segmented flow systems (determination of lactate dehydrogenase).*

simultaneously inject the same sample solution. The time difference between the arrival of both reacting plugs to the detector is fixed by the coil situated between the valves [6]. (*c*) A closed system in which, after injection by means of a valve placed outside or within the closed circuit, the sample is continuously circulated through the closed loop until complete homogenization with the enclosed carrier is achieved; a series of signals are obtained whose envelope, in the shape of a typical kinetic curve, provides interesting kinetic information on the system, in addition to the data related to the analyte concentration [7–10]. (*d*) The three most interesting possibilities of this type are: (1) splitting the injected sample into two subplugs which pass through the reactor to the cells; the subplugs are aligned with the optical path of a single detector (this is used for speciation of chromium in waters [11]). (2) Dual injection valve, which inserts the sample into two channels of different length which merge prior to the detector, and has allowed the development of a method for the differential-kinetic determination of copper and mercury [12]. (3) Splitting and confluence points which flank two reactors of different length and diameter. On the basis of this configuration, several methods have been suggested for differential-kinetic determination of cobalt and nickel in waters [13] and pyridoxal and pyridoxal-5-phosphate in human serum [14].

When two detectors are used to obtain two signals at two different times, the instrumentation needed is more expensive, but the FIA design can be very simple, as shown in figure 3(*a*) [15]. Parallel detectors can be used with the aid of a dual valve, or with single injection and splitting of the flow, as indicated in figure 3(*b*) [16 and 17].

A system which performs more complex multidetection

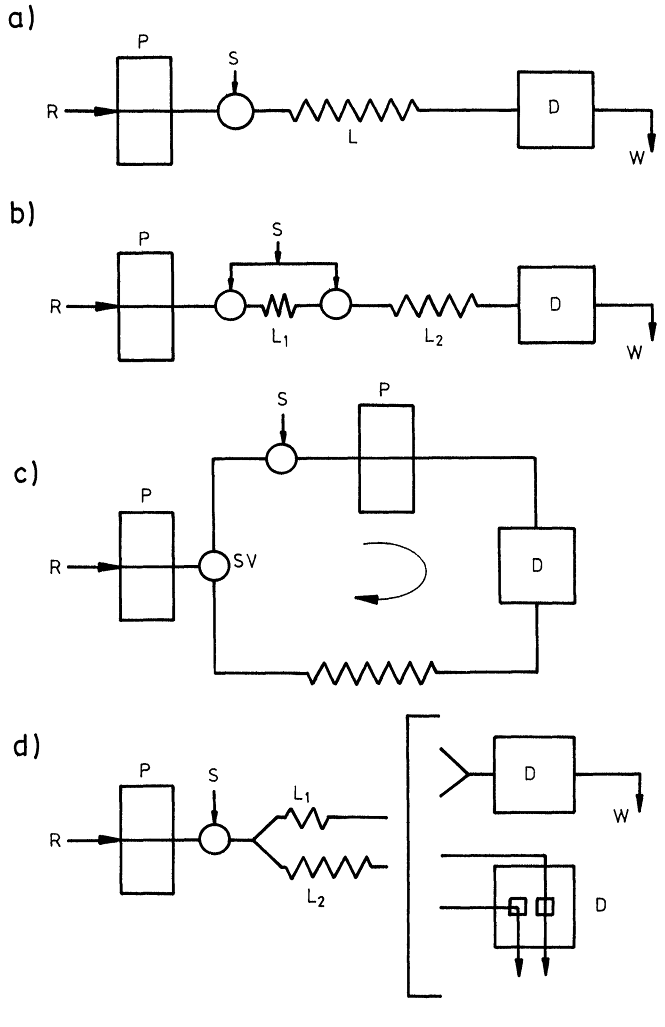

*Figure 2. FIA configurations with a single detection point used to implement kinetic methods (see text).*

than that illustrated in figure 2($c$) has been described by Hooley and Dessy [18]. It consists of a quartz reactor tube which incorporates a series of independent detection units, each of which has a LED and a photodetector, thereby allowing all of the system's kinetic data (absorbance-time) to be captured. The system is adequate for both calculation of rate constants and for kinetic determinations.

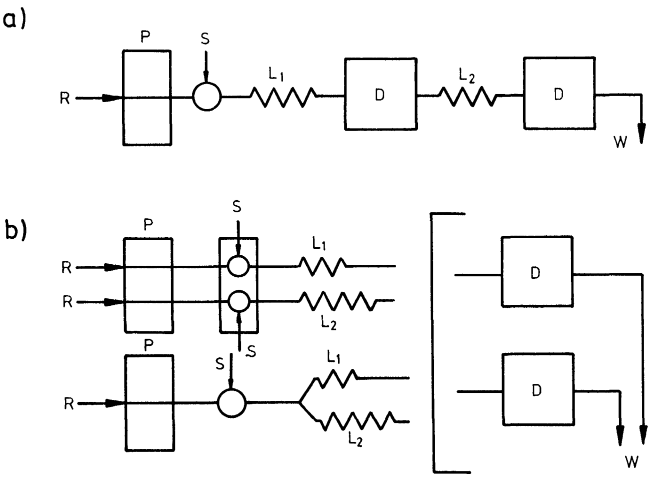

*Figure 3. FIA configurations with two detection points intended to apply kinetic methods. (a) series detectors; (b) parallel detectors.*

Stopped-flow techniques (although they should not strictly be included in this report) are briefly discussed because of their relationship to flow systems (not continuous flow). Figure 4($a$) shows the scheme of an instrument used by Malmstadt *et al.* [19] to develop conventional stopped-flow methods. The sample and reagent syringes instantaneously and simultaneously inject their contents into the system, which is halted by the stop syringe in order to start measurements. The radiation path can be parallel or perpendicular to the stream. Modifications to the instrument include mini or microcomputer control [20–23]. It can be used both for rate methods involving fast or slow reactions and for equilibrium methods. In general, the instrumentation required is relatively sophisticated and expensive.

The FIA technique also permits, with very cheap and simple instrumentation, the development of stopped-flow methods for reactions with half-lives of at least 10–20 s. There are two possible configurations (figure 4[$b$]) for developing these methods. In both cases a timer is used to control the injection system, in conjunction with either a peristaltic pump to stop the flow when the reacting plug reaches the flow-cell, or a diverting valve located prior to the detector which sends it to waste, so that the flow remains stopped from this point onwards. The pump works continuously in this case. Both configurations provide recordings such as those shown in figure 4($b$), in which the three intervals characteristic of the stopped-flow methods can be distinguished: Delay time ($\Delta t_1$), stop time ($\Delta t_2$) and washing time ($\Delta t_3$). The recordings correspond to the injection of a dye (2$a$) or to chemical systems with different reactions-rates (2$b$, 2$c$, 2$d$), as compared with the normal technique without interruptions. The FIA/stopped-flow mode offers two important advantages over conventional FIA; namely increased sensitivity and selectivity [24 and 25].

Nonsegmented continuous flow configurations with sample aspiration were originally suggested by Blaedel and Hicks for kinetic determinations. Figure 5($a$) shows the flowing system utilized for enzymatic determination of glucose. The sample is continuously mixed with the reagent and successively attains the two cells of a double-beam spectrophotometer, which monitors the absorbance difference at the two measurement times, the interval between measurements being determined on the intercell delay line [26]. The scheme in figure 5($b$) was also proposed by these authors for the determination of the activity of glutamic oxalacetic transferase. It is a *sui*

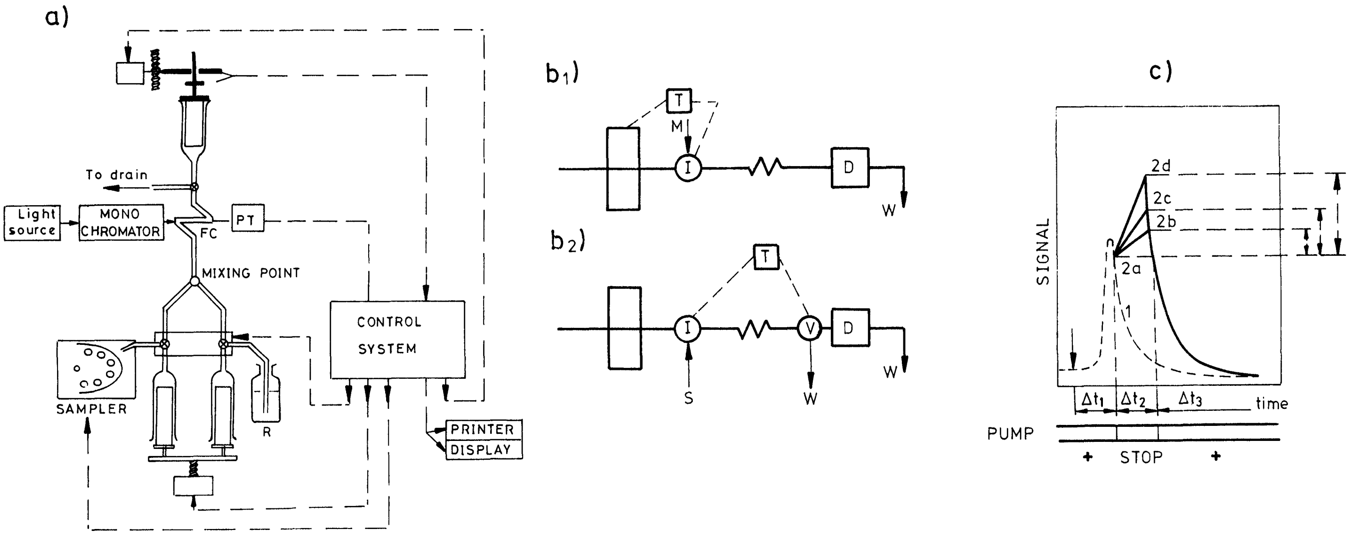

*Figure 4. (a) Conventional instrument for developing stopped-flow methods. (b) Manifolds used to implement FIA/stopped-flow methods. (b.1) With stopping of the flow; (b.2) with a diverting valve. (c) Recordings* (see text).

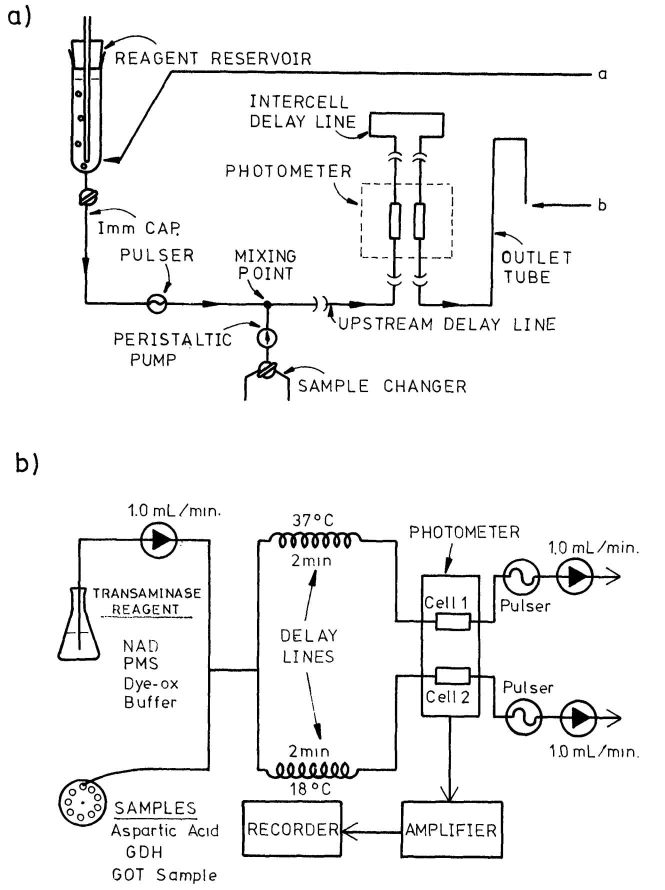

*Figure 5. Schemes of segmented flow without injection. (a) Determination of glucose: two signal measurement at two different times. (b) Determination of transaminases: two simultaneous measurements in two portions of sample at a different rate arising from difference in temperature in the reactors.*

*generis* kinetic method since measurements are based on the difference between two values of the analytical signal, a difference which does not arise from a time difference between them, which would result in a greater extent of reaction, but from a temperature difference, which results in a different reaction rate in the subsystems through which the sample-reagents mixture circulates. The configuration involves splitting of the flow after sample-reagent mixing and keeping the coils at different temperatures in such a way that when each subplug attains its respective flow-cell, the reaction has developed to a different extent, resulting in an absorbance increase which is actually the parameter measured [27].

### Acknowledgement

The authors wish to acknowledge support for this research from the Ministerio de Educación y Ciencia, through a grant from CAICyT No. 2012-83.

### References

1. Mottola, H. A., *Quím. Anal.*, **II** (1983), 3.
2. Pardue, H. L., *Quím. Anal.* II (1983), 24.
3. Skeggs, L. T., *American Journal of Pathology*, **28** (1957), 311.
4. Valcárcel, M., Luque de Castro, M. D., Linares, P. and Fernández, A., *I Symposium on Kinetics in Analytical Chemistry* (Córdoba, Spain, 1983).
5. Fernández, A., Luque de Castro, M. D. and Valcárcel, M., *Analytical Chemistry* (in press).
6. Fernández, A. (Private communication).
7. Ríos, A., Luque de Castro, M. D. and Valcárcel, M., *Analytical Chemistry*, **57** (1985), 1803.
8. Ibid. *Journal of Chemical Education* (in press).
9. Ruz, J., Ríos, A., Luque de Castro, M. D. and Valcárcel, M., *Talanta* (in press).
10. Ríos, A., Luque de Castro, M. D. and Valcárcel, M., *Analytical Chemistry*, **179** (1986) (in press).
11. Ruz, J., Ríos, A., Luque de Castro, M. D. and Valcárcel, M., *Analytica Chimica Acta* (in press).
12. Lázaro, F., Luque de Castro, M. D. and Valcárcel, M., Fresenius, Z., *Analytical Chemistry* **320** (1985), 128.
13. Fernández, A., Luque de Castro, M. D. and Valcárcel, M., *Analytical Chemistry*, **56** (1984), 1146.
14. Linares, P., Luque de Castro, M. D. and Valcárcel, M., *Analytical Chemistry*, **57** (1985), 2101.
15. Dahl, J. H., Espersen, D. and Jensen, A., *Analytica Chimica Acta*, **105** (1979), 327.
16. Espersen, D. and Jensen, A., *Analytica Chimica Acta*, **108** (1979), 241.
17. Betteridge, D. and Fields, B., Fresenius, Z., *Analytical Chemistry*, **314** (1983), 386.
18. Hooley, D. J. and Dessy, R. E., *Analytical Chemistry*, **55** (1983), 313.
19. Javier, A. C., Crouch, S. R. and Malmstadt, H. V., *Analytical Chemistry*, **41** (1969), 239.
20. O'Keefe, K. R. and Malmstadt, H. V., *Analytical Chemistry*, **47** (1975), 707.
21. Krottinger, D. L., McCracken, M. S. and Malmstadt, H. V., *American Laboratory*, March **9**(3) (1977), 51.
22. Mieling, G. E., Taylor, R. W., Hargis, L. G., English, J. and Pardue, H. L., *Analytical Chemistry*, **48** (1976), 1686.
23. Breckwith, P. M. and Crouch, S. R., *Analytical Chemistry*, **44** (1972), 221.
24. Lázaro, F., Luque de Castro, M. D. and Valcárcel, M., *Analytica Chimica Acta*, **165** (1984), 177.
25. Linares, P., Luque de Castro, M. D. and Valcárcel, M., *Analytica Chimica Acta*, **161** (1984), 257.
26. Blaedel, W. J. and Hicks, G. P., *Analytical Chemistry*, **34** (1962), 382.
27. Hicks, G. P. and Blaedel, W. J., *Analytical Chemistry*, **37** (1965), 354.

